# An Old Comrade's Association

**Published:** 1920-11-20

**Authors:** V. C. C. Collum


*To the Editor of* The Hospital.

### "An Old Comrade's Association."

*To the Editor of* The Hospital.

Sir,—I have to thank you for so kindly inserting the few remarks about our Royaumont Association, but hasten to disclaim the honour of being the Hon. Secretary (talented or otherwise!). I am just one of the rank and file who happens to have brought up this scheme. Our Hon. Secretary is our late "second in command," Miss Ruth Nicholson, M.B., Ch.B., who was the "right-hand man" of our C.M.O., Miss Tulus, M.S. (now our President), and who was one of the first to arrive in December 1914, and the last to leave in the spring of 1919. With renewed thanks, I am, yours faithfully,

V. C. C. Collum.

